# Sleep, psychological symptoms, and cannabis use before, during, and after COVID-19 “stay-at-home” orders: a structural equation modeling approach

**DOI:** 10.1186/s42238-025-00269-9

**Published:** 2025-03-24

**Authors:** Nicole P. Bowles, Sean P. M. Rice, Joey Hebl, Alicia V. Stewart, Steven A. Shea

**Affiliations:** Oregon Institute of Occupational Health Sciences, Oregon Health and Sciences University, 3181 SW Sam Jackson Park Rd., L606, Portland, Oregon 97239-3098 USA

**Keywords:** Marijuana, Lockdown, Coronavirus, Substance use, Drug use, Longitudinal

## Abstract

**Background:**

Given the frequent use of cannabis for sleep and mental health symptoms, we aimed to prospectively examine the reciprocal relationship between sleep, mental health, and cannabis use from before, during, and 1 year after the implementation of COVID-19 stay-at-home orders.

**Methods:**

Five hundred thirty-four young adults (21–34 years old) from Oregon and Washington States who previously completed a survey on their sleep and cannabis use prior to stay-at-home orders (T0), were followed up after initial stay-at-home orders were in place for approximately two months (T1), and one year later (T2), to reassess sleep and cannabis use. Sleep measures included the presence of sleep trouble [yes/no], and weekday and weekend sleep duration. The Cannabis Use Disorders Identification Test Revised (CUDIT-R) was used to assess past-six-month cannabis use. The follow up surveys additionally measured mental health symptoms and other health behaviors. We applied cross-lagged panel models to assess the association between cannabis use and sleep at all three time points. Multivariate parallel latent change score models were used to assess associations between changes in cannabis use, sleep, mental health symptoms, and other behavioral factors from T1 to T2.

**Results:**

For the cross-lagged models, reporting sleep trouble at T0 was associated (β = 0.18, *p* < 0.05) with higher CUDIT-R scores at T1, however this relationship did not hold from T1 to T2. CUDIT-R scores were not associated with sleep trouble from T0 to T1, however CUDIT-R at T1 had a positive association (β = 0.35, *p* < 0.05) with sleep trouble at T2. The two-wave latent change score model, indicated that change in sleep trouble between T1 and T2 was positively associated with changes in depression (*r* = 0.37, *p* < 0.05) and anxiety (*r* = 0.40, *p* < 0.05) across this period. No significant correlation was found between changes in cannabis use and changes in sleep trouble.

**Conclusions:**

Our findings suggest an inconsistent bidirectional link between sleep trouble and cannabis use. Only sleep trouble, and not cannabis use, predicted mental health measures. These associations lend support for a holistic approach to designing and implementing public health measures during a global pandemic.

**Supplementary Information:**

The online version contains supplementary material available at 10.1186/s42238-025-00269-9.

## Introduction

In the United States, Oregon and Washington were among the first states to implement “stay-at-home” orders following the declaration of the coronavirus disease 2019 (COVID-19) as a pandemic by the World Health Organization on March 11th, 2020. Over the next two years, these states used various public health measures to manage disease outbreaks through implementation and modification of social distancing measures, mask mandates, vaccination requirements, non-essential business and school closures, and restrictions on international travel. Although these measures were intended to minimize the spread of COVID-19, they also resulted in unemployment and financial uncertainty for many Americans. By April 2020, unemployment rose to 14.9% and 16.3% in Oregon and Washington, respectively, not returning to pre-pandemic levels until November, 2021 (Edwards et al. 2022). These measures increased levels of social isolation; made for stressful home environments that involved online/hybrid school and work-from-home arrangements; precluded most public recreational activities; and contributed to a national increase in mental health problems including anxiety and depression (Brown et al. 2020; Shah et al. 2021; Niedzwiedz et al. 2021; Czeisler et al. 2020).

Initial analyses following work-from-home requirements and school closures reported a mix of longer sleep times and reduced social jet lag (Alfonsi et al. 2021; Wang et al. 2021; Yuan et al. 2022; Brandão et al. 2021; Wesley et al. 2023). However, subsequent epidemiological studies reported that increased levels of stress, anxiety, and social isolation during periods of lockdown were associated with increased perceived sleep disturbances, sleep latency, and insomnia symptoms (Kokou-Kpolou et al. 2020; Stanton et al. 2020; Son et al. 2020; Marelli et al. 2021; Alimoradi et al. 2021; Martínez-de-Quel et al. 2021). The psychological burden of COVID-19 and related public-health measures were also associated with varied changes in substance use behaviors, including the use of cannabis (Vanderbruggen et al. 2020; Niedzwiedz et al. 2021; Czeisler et al. 2020; Bonar et al. 2021; Roberts et al. 2021). Prior to COVID-19, the use of cannabis in the US had steadily increased as a result of increased legalization and a reduced perception of risk (Hasin 2018). Cannabis is frequently used to manage stress and sleep disturbances, though evidence of objective improvement in the latter is inconsistent and deviates significantly based on the frequency of cannabis use (Babson et al. 2017; Bowles et al. 2017; Spradlin and Cuttler 2019; Ried et al. 2023; Walsh et al. 2021; Gilman et al. 2022; Cooke et al. 2023). Findings on the use of cannabis during the pandemic are mixed, with a majority of reports using cross-sectional approaches that relied upon recall of past cannabis use, thus introducing recall bias (Brotto et al. 2021; Imtiaz et al. 2021; Busse et al. 2021; Benschop et al. 2021; Mehra et al. 2023; Knell et al. 2020; Boehnke et al. 2021; Armour et al. 2022). Further, it is unclear whether those who experienced increased psychological distress and sub-optimal sleep during the pandemic also had changes in their cannabis use. The present study aims to prospectively examine the relationship between sleep, mental health, and cannabis-use across the pandemic.

We assessed a sample of young adults from Oregon and Washington with and without prior histories of cannabis use who were previously screened for a study on the acute effects of tetrahydrocannabinol (THC). Participants completed follow-up surveys just after (May, 2020) and approximately a year (May, 2021) following the initial and major stay-at-home orders issued in both states. We hypothesized, by looking at before, during, and after stay-at-home order implementation, that structural equation modeling would reveal reciprocal relationships between cannabis-use with total time in bed (TTB) and sleep quality. Additionally, we hypothesized that changes in cannabis use from the implementation of stay-at-home orders until a year later would be associated with other health measures, including self-reported stress, anxiety, and depression.

## Methods

### Study participants and design

We recruited 534 healthy young adults (21–34 years old) with and without prior histories of cannabis use who had previously completed a screening survey for the “Effect of tetrahydrocannabinol (THC) on sleep in humans” study in Portland, Oregon, and had at that time consented to have their information saved for future contact. These baseline data, starting in July 2018, are here referred to as T0 or pre-stay-at-home orders. This population was invited to participate in a follow-up study to report their cannabis habits during the stay-at-home orders, as well as assess mental health symptoms and health behavior outcomes. This follow up, here referred to as T1, started on May 19, 2020, approximately two months after implementation of stay-at-home orders and before the initial re-opening of major cities. Individuals who did not include an email address; reported having diabetes, cancer, or a cardiovascular event (e.g., heart failure, angioplasty, heart surgery); were on blood pressure medications; and/or reported that they were pregnant were excluded from the follow-up online survey (*n* = 194). Upon completion of this survey, participants were randomly selected (1-in-10) to win a $10–25 Amazon gift card. Participants who completed stay-at-home surveys and consented for follow up were invited to complete surveys one year later (May 2021, here referred to as T2) and were compensated with a $25 gift card. After the initial email invitation, participants were sent up to two additional invitation reminders. A research assistant also called survey non-responders one-time and sent the survey to a new email address if provided during the phone call. Study data were collected and managed using REDCap (Research Electronic Data Capture) hosted at Oregon Health & Science University (Harris et al. 2009, 2019). REDCap is a secure, web-based software platform designed to support data capture for research studies, providing 1) an intuitive interface for validated data capture; 2) audit trails for tracking data manipulation and export procedures; 3) automated export procedures for seamless data downloads to common statistical packages; and 4) procedures for data integration and interoperability with external sources. All participants completed a consent form before filling our surveys and were provided contact numbers of SAMHSA disaster and national helplines. The study was approved by Oregon Health & Sciences University’s Institutional Review Board (#18052).

### Study measures

#### Demographics

During the initial screen participants provided their gender and date-of-birth. In follow-up surveys, additional questions were added to record each participant’s race (White, Black/African American, Asian, American Indian/Alaskan Native, Native Hawaiian/Pacific Islander); ethnicity (hispanic/latino or not hispanic/not latino); income (< $15,000; $15,000-$44,999; $45,000-$89,999; $90,000-$150,000; > $150,000); and formal education level (< high school, high school, some college, ≥college graduate). Additionally, participants were asked if they were currently employed, and when/if employed, if, during the stay-at-home orders, they were required to go into the workplace, worked virtually, or worked a hybrid model.

#### Cannabis use

The 8-item Cannabis Use Disorders Identification Test Revised (CUDIT-R) was used to estimate disordered cannabis use (Adamson et al. 2010). The CUDIT-R assesses cannabis use within the past 6-months. Responses were summed to generate a total score, providing a continuous index of cannabis misuse (range 0–32). Scores ≥8 are indicative of hazardous use and scores ≥12 are indicative of disordered use. When examining participant characteristics, the survey’s initial question “How often do you use cannabis?” (Never, monthly or less, 2–4 times a month, 2–3 times a week, or 4 or more times a week) was used to assess the frequency of cannabis use.

#### Sleep

Participants were asked to separately provide their average times in and out of bed on weekdays and weekends (e.g., “Over the past month, what is your usual bedtime on weekdays?”). From these values total time in bed (TTB) was calculated for weekdays and weekends. Sleep quality, measured as “Sleep Trouble” was assessed from a single “yes/no” question (“Over the past month, have you had any trouble with your sleep?”).

#### Psychological symptoms

Patient-Reported Outcomes Measurement Information System (PROMIS) 4-item questionnaires were used to assess symptoms of depression and anxiety. PROMIS raw scores are normalized (t-scores) to a standard population distribution with a mean of 50 and a standard error of 10. Higher t-scores indicate higher levels of the health concept. For example, higher depression scores indicate greater depression symptoms. The 10-item Perceived Stress Scale (PSS) was used to measure levels of stress (Cohen et al. 1983). The scale ranges from 0 to 40 with a higher score indicating a higher stress level.

#### Health behaviors

The use of tobacco was posed at all 3 time points (T0, T1 and T2): “Are you currently using any tobacco products?” (yes/no). Additionally, at collections T1 and T2, the following questions were asked to measure alcohol use and diet. For alcohol there were three items: “For the last 30 days on average, how many days per week do you drink alcohol?” (0–7 days), “For the last 30 days on a typical day when you drink, how many drinks do you have?” (continuous numeric response allowed), and “For the last 30 days what is the maximum number of drinks you had on any given occasion?” (continuous numeric response allowed). We applied a confirmatory factor analysis on these alcohol items to identify a latent “alcohol use” variable. From this model, we extracted a factor score (i.e., a summary alcohol use Z-score for each participant) and used this value for analyses. For diet, we summed the average number of daily fruits and vegetables consumed with two items, “How many servings of fruit do you eat each day?” and “How many servings of vegetables do you eat each day?” (continuous numeric responses allowed for both).

### Statistical analysis

All main variables were assessed descriptively at each timepoint. We compared baseline age, sex, CUDIT-R scores, and sleep trouble between dropouts and completers (i.e., individuals who provided data at all timepoints) via independent samples t-tests and chi-square. Unequal variance corrections for degrees of freedom, and Fisher’s exact test were applied in cases on unequal variances (t-test) and low cell counts (chi-square), respectively. We also used repeated measures ANOVAs (continuous variables, three timepoints), paired samples t-tests (continuous variables, two-timepoints), Cochran’s Q (categorical variables, three timepoints), and McNemar’s test (categorical variables, two timepoints) to compare the valid (i.e., observed) values of CUDIT-R, alcohol use, sleep trouble, fruit and vegetable servings, sleep duration, and psychological health across time.

To address our primary aim, we applied cross-lagged panel models, which allow for the examination of the reciprocal directional effects with longitudinal data (Duncan 1969; Cole and Maxwell 2003). In these models, we evaluated sleep (weekday or weekend TTB or sleep trouble, respectively) and CUDIT-R score at each of the three time points. Each variable at T1 was regressed on both variables at T0, and each variable at T2 was regressed on both variables at T1, respectively. Models with weekday or weekend TTB were estimated linearly with restricted maximum likelihood estimation. Because sleep trouble was a dichotomous endogenous variable (i.e., outcome), diagonal weighted least squares estimation and theta parameterization was used to identify that model (Savalei 2021). This type of estimation and parameterization results in probit regression coefficients for sleep trouble and linear regression coefficients for cannabis (as a continuous variable). In other words, raw coefficients in a probit model represent the effect of a predictor on the change in outcomes’ Z-scores, which are transformed into the probability of the event occurring (Muthén 1979). Model fit was evaluated using chi-square, comparative fit index (CFI) and standardized root mean square residual (SRMR). Accepted cutoffs of CFI > 0.90 and SRMR < 0.08 were used to infer acceptable fit of the model (DiStefano et al. 2018). Significance of regression paths was set a priori at 0.05.

To address our secondary aim, the examinations of changes in self-reported sleep quality and self-reported stress measures, we only had data available from T1 and T2. With these two periods of data, we generated multivariate parallel latent change score (LCS) models with sleep (either weekend/weekday TTB or sleep trouble), stress, anxiety, depression, CUDIT-R, fruit and vegetable consumption, and alcohol use. LCS models apply structural equation modeling to identify variable change as a single latent factor loading onto specific waves of interest for change, which can then be correlated with other change variables (Grimm et al. 2016; Newsom 2015). In the present study, we identified a change structure for all of the variables listed above loading onto their respective T1 and T2 observed variables. Only T1 and T2 were used for this analysis, since the psychological symptoms were only measured during those periods (not during T0). Model specifications including parameter constraints were made in accordance with published guidelines for two-wave LCS models (Grimm et al. 2016; Newsom 2015). Only covariances were estimated to avoid convergence issues found in directional effects models. The same estimation, parameterization, and model fit inferences from the previous analyses were used here. Significance of the correlations among latent change scores was set a priori at 0.05.

## Results

### Descriptive results

A total of 190 participants from the original T0 population of 534 (36%) completed the follow-up survey from May–June 2020 (T1), and 126 (24% of T0 sample; 66% of T2 population) completed the surveys May–June 2021 (T2). Dropout comparisons suggested that individuals who participated in all the surveys had no significant differences in demographic characteristics at baseline from those participants who dropped out. However, both sex and total CUDIT-R score were approaching significantly different, such that females were slightly more represented in the completers (64%) compared to the dropouts (54%; *p* = 0.05), and dropouts had a slightly higher total CUDIT-R score (mean difference = 1.4; *p* = 0.06). Descriptive statistics of the study population pre-, during-, and post-COVID-19 stay-at-home orders are displayed in Table 1 with the exception of variables only collected during stay-at-home orders. There were no observed differences over time in terms of weekday sleep TTB, emotional support, and alcohol use (all *p* > 0.05). The majority of participants reported using cannabis four or more times per week, and 29%−36% scored 12 or greater on the CUDIT-R, indicating possible disordered cannabis use (Adamson et al. 2010). Both the frequency of cannabis use (F = 4.4, *p* < 0.05) and the overall CUDIT-R score (F = 4.4, *p* < 0.05) significantly changed over time, such that the frequency decreased from T0-T2, but the overall CUDIT-R score increased from T0-T1 and T0-T2, respectively (pairwise ps < 0.05). On average, the population self-reported TTB greater than eight hours across all time points on both weekdays and weekends. The mean weekend TTB did significantly decline over time (F = 3.2, *p* < 0.05), but only by 0.3 h from T0-T2. The dichotomous assessment of sleep trouble significantly differed across time (Q = 14.9, *p* < 0.05) with the largest percentage of sleep trouble reported during the initial stay-at-home orders (T1). During this stay-at-home period, the mean reported score for perceived stress was 21.0 ± 3.7 (score range 0–40) suggesting moderate to high levels of stress, but this significantly decreased by T2 (*p* < 0.05). Moderate levels of depression (54.3 ± 9.3) and anxiety symptoms (57.1 ± 9.2) occurred during stay-at-home orders (T1) and anxiety symptoms significantly decreased at T2, one year later (all *p* < 0.05).

**Table 1:** Participant characteristics at baseline and study follow-up

	Time 0 Pre-COVID-19 *n* = 534 mean ±SD or n (%)	Time 1 COVID-19 Stay-at-Home Orders (May–June 2020) *n* = 190 mean ±SD or n (%)	Time 2 1 year follow up (May–June 2021) *n* = 126 mean ±SD or n (%)
Gender (Female)	301 (56%)	115 (61%)	79 (62%)
Age	26.6 ± 3.8	27.5 ± 3.9	28.6 ± 3.7
Education			
< high school	n/a	1 (1%)	0 (0%)
high school	n/a	18 (9%)	6 (5%)
some college	n/a	54 (28%)	29 (23%)
≥college graduate		117 (62%)	91 (72%)
Employment Status			
Working from home	n/a	190 (35%)	38 (30%)
Working and required to go into workplace	n/a	62 (33%)	63 (50%)
Unemployed	n/a	49 (26%)	11 (9%)
Student	n/a	13 (7%)	14 (12%)
Income			
< $15,000	n/a	32 (17%)	17 (13%)
$15,000-$44,999	n/a	74 (39%)	45 (36%)
$45,000-$89,999	n/a	52 (27%)	30 (24%)
$90,000-$150,000	n/a	28 (15%)	25 (20%)
> 150,000	n/a	4 (2%)	9 (7%)
Latent factor score of alcohol consumption/week (days)^a^	n/a	−0.09 (−0.84, 0.56)	−0.06 (−0.63, 0.56)
Current tobacco use (% yes)^a^	64 (12%)	18 (9%)	14 (11%)
Fruit and vegetable consumption	n/a	4.8 ± 2.5	4.3 ± 2.2
Cannabis Use Frequency			
Never	90 (17%)	35 (18%)	26 (21%)
Monthly or less	52 (10%)	18 (10%)	18 (14%)
2–4 times a month	34 (6%)	17 (9%)	14 (11%)
2–3 times a week	79 (15%)	25 (13%)	12 (10%)
4 or more times/week	278 (52%)	95 (50%)	56 (44%)
CUDIT-R Score (0–32)	8.6 ± 7.0	8.8 ± 6.8	8.2 ± 6.9
CUDIT-R ≥12	191 (36%)	63 (33%)	36 (29%)
Average Weekday Total Time in Bed (hours)	8.3 ± 1.3	8.3 ± 1.2	8.1 ± 1.1
Average Weekend Total Time in Bed (hours)	8.7 ± 1.3	8.7 ± 1.4	8.5 ± 1.4
Trouble Sleeping (% yes)	233 (44%)	122 (64%)	70 (56%)
Perceived stress (0–40)	n/a	21.0 ± 3.9	20.2 ± 3.9
Depression Symptoms^b^	n/a	54.2 ± 9.0	52.5 ± 9.1
Anxiety Symptoms^b^	n/a	56.7 ± 9.1	54.2 ± 9.6

^a^Values are in Median and Interquartile Range because factor scores are standardized values

^b^T-scores (normalized values) from Patient-Reported Outcomes Measurement Information System (PROMIS): depressive and anxiety symptoms, higher score indicate high symptom severity

### Structural equation models

**Fig. 1:**
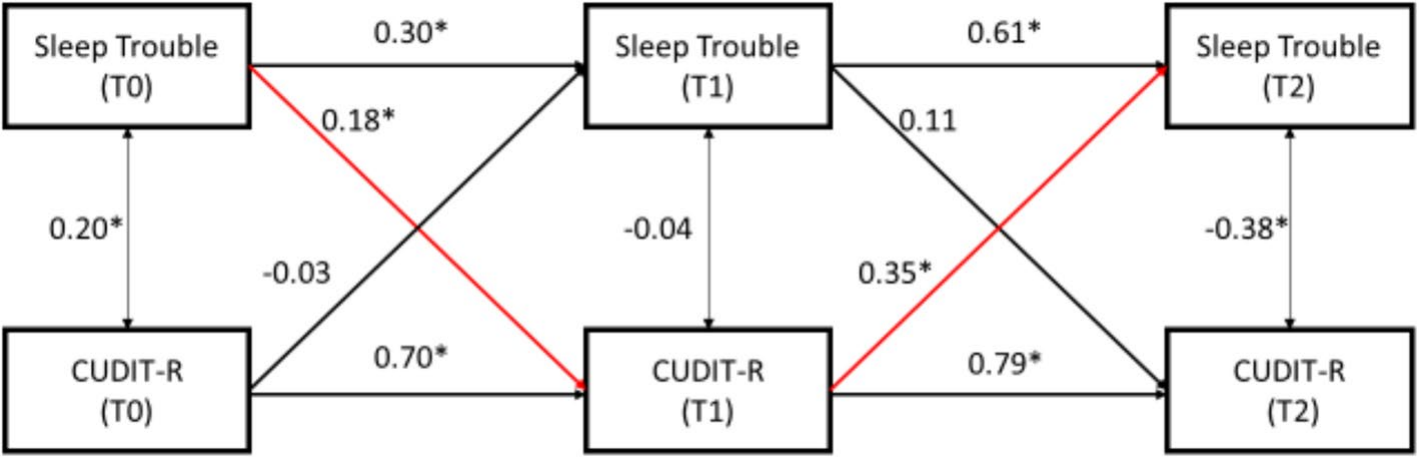
Cross-Lagged Panel Model comparing sleep trouble with CUDIT-R scores for the three time periods. Sleep Trouble (a dichotomous “Yes”/”No” variable) andCUDIT-R (Cannabis Use Disorders Identification Test Revised, a continuous variable) were determined at each time point (T0, T1, and T2). Significance determined as *p* < 0.05 and labeled in the figure with the “*” symbol. Significant, cross-path arrows labeled red, while non-significant or non-relevant arrows labeled with black

Figure 1 represents the cross-lagged panel model for sleep trouble and CUDIT-R score. Model fit was good, χ^2^ = 4.16, *p* > 0.05; CFI = 0.99; SRMR = 0.08. Reporting sleep trouble at T0 (pre-COVID stay-at-home orders) was associated (β = 0.18, *p* < 0.05) with higher CUDIT-R scores (i.e., more hazardous levels of cannabis use) at T1 (during COVID stay-at-home orders), controlling for T0 CUDIT-R score. Additionally, CUDIT-R scores at T1 had a positive association (β = 0.35, *p* < 0.05) with sleep trouble at T2 (sleep trouble 1-year post COVID stay-at-home orders), suggesting that increased CUDIT-R scores during stay-at-home orders (T1) led to an increased probability of having sleep trouble one year after stay-at-home orders (T2), after controlling for T1 sleep trouble status. The cross-paths (i.e., CUDIT-R at T0 to sleep trouble at T1; sleep trouble at T1 to CUDIT-R at T2) were not significant (*p* > 0.05), indicating support for the former paths’ directionality. In the model for weekday TTB, no cross-paths were significant (supplemental Fig. 1). However, increased weekend TTB at T0 was associated with increased CUDIT-R scores at T1 (β = 0.13, *p* < 0.05) (Supplemental Fig. 2). No other cross-paths were significant for weekend TTB and CUDIT-R score. Additionally, all autoregressive paths (e.g. looking at the coefficient for CUDIT-R between T0 and T1, and again between T1-T2, etc.) were significant in the expected direction (e.g. the positive, significant correlation between CUDIT-R at T0 and T1 suggest that having elevated CUDIT-R scores at T0 is associated with elevated CUDIT-R scores at T1, etc.).

**Fig. 2:**
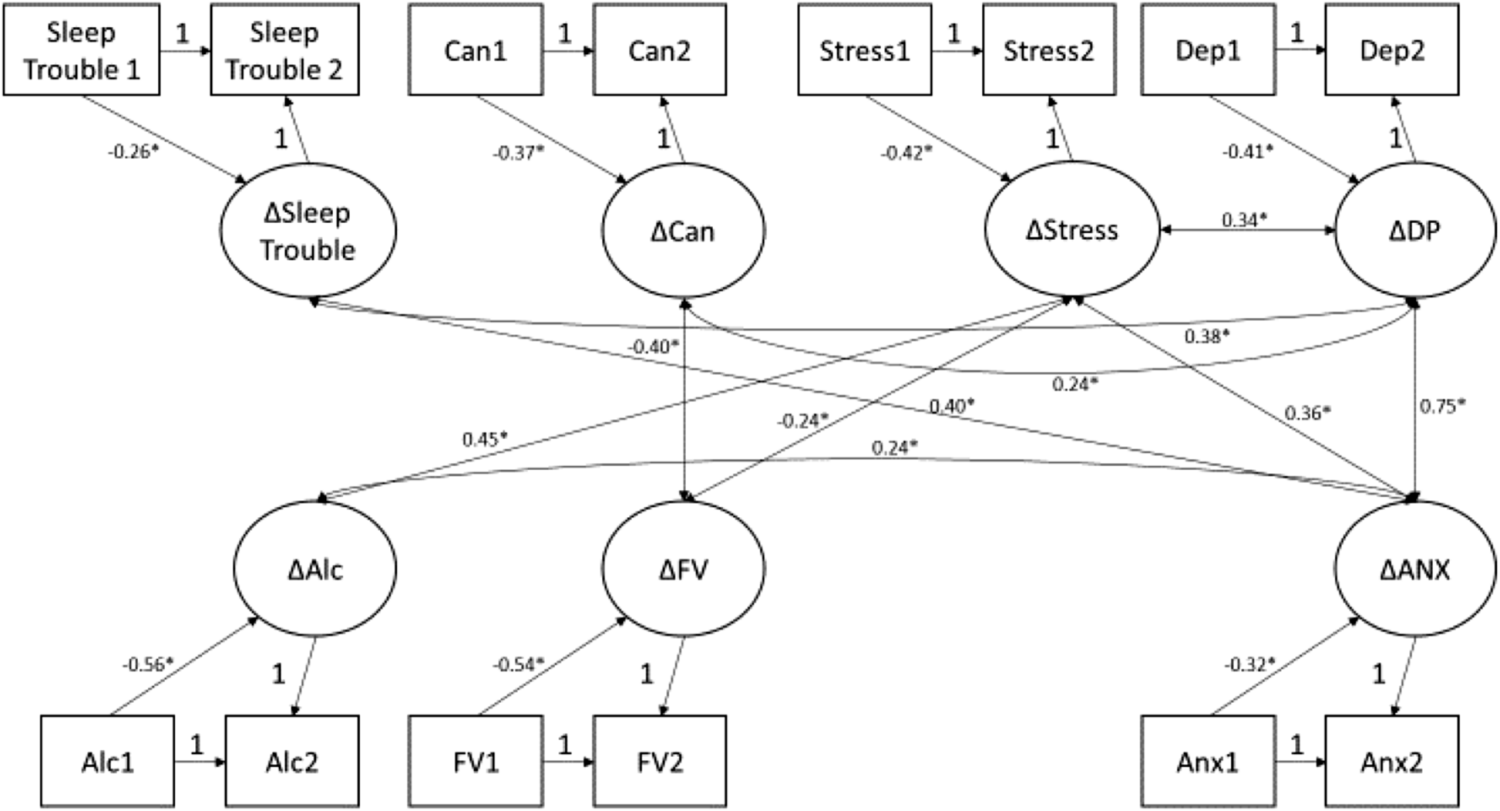
Latent Change Score Model assessing health-behavior metric associations between T1 (stay-at-home measures) and T2 (1 yr after stay-at-home measures). “Sleep Trouble” = sleep trouble, “Can” = CUDIT-R score, “Stress” = stress score from perceived stress scale (PSS), “Dep” = depression score from PROMIS questionnaire, "Anx” = anxiety score from PROMIS questionnaire, “Alc” = alcohol use as latent factor score, “FV” = consumption of fruits/vegetables as continuous numeric value. Directional arrows and associated path coefficients are shown for only those comparisons that resulted in a significant relationship (*p* < 0.05 indicated by “*”)

Figure 2 represents the two-wave multivariate parallel latent change score model reflecting changes between T1 and T2. Model fit was acceptable, χ^2^ = 73.25, *p* < 0.05; CFI = 0.93; SRMR = 0.07. Changes in depression, anxiety, and stress were all positively associated (*p* < 0.05), indicating that increases in one (e.g., depression) were associated with increases in another (e.g., anxiety) between time points. Change in CUDIT-R was negatively associated (*r* = −0.40, *p* < 0.05) with change in fruit and vegetable consumption (i.e., increases in CUDIT-R between T1 and T2 were associated with decreases in fruit and vegetable consumption), and positively associated with depression (*r* = −0.24, *p* < 0.05). Finally, change in sleep trouble status was positively associated with changes in depression (*r* = 0.37, *p* < 0.05) and anxiety (*r* = 0.40, *p* < 0.05), indicating that going from no sleep trouble to having sleep trouble (dichotomous variable) was also associated with increasing levels of depression and anxiety. No significant correlations were found between changes in CUDIT-R and sleep trouble, nor weekday or weekend TTB (supplemental Figs. 3 and 4, respectively).

## Discussion

This study evaluated the direction of the association between cannabis use behaviors, assessed via CUDIT-R scores, TTB, and sleep trouble before, during, and after COVID-19 stay-at-home orders. Additionally, it assessed the association between changes in CUDIT-R in relation to stress, anxiety, depression, alcohol use, diet, and sleep during stay-at-home orders and after stay-at-home orders. The results of our cross-lagged panel model partially supported our hypothesis that higher cannabis dependence during COVID-19 stay-at-home orders predicts increased trouble sleeping a year later. However, only the converse was true when looking at the time points prior to and during stay-at-home orders. That is to say, sleep troubles prior to lockdown predicted CUDIT-R scores during lockdown, but CUDIT-R scores prior to lockdown did not predict sleep troubles during lockdown. Though inconsistent, these findings are suggestive of a reciprocal relationship between sleep troubles and cannabis use behaviors, consistent with reports on the use of cannabis for self-medicating sleep troubles (Babson et al. 2017; Bowles et al. 2017; Ciesluk et al. 2023; Llewelyn-Williams and Mykota 2023). No relationships were found for weekday TTB and CUDIT-R scores across all three time points. The lack of associations between TTB and other measures may be partly due to the formers insufficient sensitivity for assessing sleep, as in sleep duration or sleep efficiency—assessed with scales such as the Pittsburg Sleep Quality Index (PSQI) (Buysse et al. 1989)—which take into consideration sleep latency and wake-after-sleep-onset, both of which can adversely impact sleep. This may help explain the counter-intuitive finding of around half of participants, across the three time points, endorsing sleep troubles while also reporting an average TTB of greater than 8 h, which is the recommended sleep duration for adults.

Based on our LCS model, changes in reported sleep trouble from during stay-at-home orders to after stay-at-home orders was positively associated with symptoms of depression and anxiety, but not to changes in CUDIT-R. Changes in CUDIT-R was positively associated with depression but not with sleep and stress.

Reports on the changes in cannabis use behaviors during lockdown measures from a number of cross-sectional studies evaluating self-reported cannabis use are mixed, with the directional change of cannabis use from before to during lockdown measures found to be insignificant (Vanderbruggen et al. 2020), equivocal (Salles et al. 2021; Mehra et al. 2023; Busse et al. 2021), and/or vary greatly across the respondents (Zajacova et al. 2020; Mehra et al. 2023; Benschop et al. 2021; Yousufzai et al. 2022; Tholen et al. 2022). A repeat-measures study among young adults in Washington State (assessed in January 2020 and once more April/May 2020) with an approach and a population similar to that of the present study found no change in cannabis use (Graupensperger et al. 2021). This was also the case for a sustained cannabis use study in Canada which, even across six repeated cross-sectional surveys conducted later in the pandemic (May to December 2020), found no change in the daily trends of cannabis use (Imtiaz et al. 2021). Alternatively, utilizing a similar method of pre-lockdown (8–9 months prior to lockdown measures implemented on March 12, 2020 in the Netherlands) and follow-up surveys (assessed April and June of 2020),*Cousjin et. al.*found that among a population of adults residing in the Netherlands, cannabis use during lockdown increased, however, Cannabis Use Disorder symptoms did not change (Cousijn et al. 2021). Additionally, Bonar et. al, in their cross-sectional study in a population of young adults engaged in a pre-COVID-19 random-control trial on cannabis-use cessation, found that 50% of respondents had increased use during lockdown (Bonar et al. 2021), as also reported, in another study, by individuals using cannabis for medical purposes (Lake et al. 2023). The present study extends the follow-up evaluative period to a full year after the initial and most restrictive stay-at-home measures—for Oregon and Washington this was a time characterized by a cycle of phased openings and closings of social gathering businesses and spaces (e.g., restaurants and gyms) based on COVID-19 community infection rates—yet we identified no clear pattern of change in cannabis use during the pandemic.

Although the present results from our LCS model did not demonstrate a significant relationship between cannabis and stress or sleep during the pandemic, it did find a positive correlation between cannabis use and symptoms of depression. This finding adds to extensive COVID literature supporting the association between cannabis use and psycho-social factors like anxiety (Dyar et al. 2021; Yousufzai et al. 2022; Somé et al. 2022; Brotto et al. 2021; Cousijn et al. 2021), depression (Bonar et al. 2021; Dyar et al. 2021; Somé et al. 2022; Tholen et al. 2022; Busse et al. 2021; Knell et al. 2020; Brotto et al. 2021), and social isolation/loneliness (Bartel et al. 2020; Bonar et al. 2021; Somé et al. 2022; Benschop et al. 2021; Brotto et al. 2021; Vanderbruggen et al. 2020); factors which were adversely impacted by the pandemic (Khubchandani et al. 2021; Coley et al. 2022; Czeisler et al. 2020). In our LCS model, independent of cannabis use, changing depression and anxiety symptoms were also positively associated with sleep troubles during and after stay-at-home orders, in line with prior research (Marelli et al. 2021; Alimoradi et al. 2021; Martínez-de-Quel et al. 2021). Despite the frequent endorsement of cannabis use for stress management (Webb and Webb 2014; Hyman and Sinha 2009; Glodosky and Cuttler 2020), our null findings on an association between cannabis use and stress are consistent with a number of studies that fail to confirm the purported stress-relieving benefits of cannabis use (Spradlin and Cuttler 2019; Davis et al. 2024; Gobbi et al. 2019).

We did not specifically differentiate between recreational and medical cannabis use. It is worth noting that most individuals who use cannabis for medical purposes also use it recreationally (Wall et al. 2019) or have previously used it recreationally making it difficult to differentiate specific effects (Lent et al. 2024). For example, while a recent study by Lent et al. noted a dramatic improvement in health-related quality of life, inclusive of social and emotional well-being, three months after initiating a medical marijuana program, 25% of participant also noted recent recreational use (Lent et al. 2024). In studies that do differentiate between user motivations, individuals consuming cannabis for medicinal purposes consume cannabis more frequently (Metrik et al. 2018), are more likely to indicate having poor sleep and physical health (Metrik et al. 2018; Lin et al. 2016), and have lower rates of alcohol or drug use disorders compared to those consuming cannabis for recreational purposes (Lapham et al. 2023; Browne et al. 2022; Lin et al. 2016). Collectively, these findings suggest that context and purpose of cannabis use may impact outcomes and should be documented.

The finding that cannabis use decreased for significant portions of some study populations, including in the present study, seems counterintuitive given that the increased psycho-social stress of the pandemic and stay-at-home orders would typically be expected to lead to greater utilization of coping mechanisms, such as cannabis use (Sznitman et al. 2022; Vedelago et al. 2022; Bresin and Mekawi 2019; Espinoza et al. 2023; Bartel et al. 2020; Yousufzai et al. 2022; Benschop et al. 2021). The apparent decrease in use for some people may be partly due to concerns based on the well-known association of COVID-19 with lung injury (Camporota et al. 2022). Given the established connection between lung injury and smoke inhalation (including smoked cannabis) (Kaplan 2021), concerns around use and increased risk of infection and of a poorer prognosis may have prompted some people to decrease their cannabis use, and others to switch their consumption modalities (i.e. consuming more edibles) (Armour et al. 2022). Decreased opportunity for the communal consumption of cannabis (i.e., with friends, as is common in emerging and young adults) was a prominent reason provided by respondents in several studies (Benschop et al. 2021; Tholen et al. 2022; Merrill et al. 2022). Additionally, variations in one’s ability and/or comfort with accessing cannabis products during lockdown periods (e.g., concerns about social contact and risk of infection, changes in normal sources of cannabis, lack of disposable income) may also help to explain the varied changes in cannabis use (Vanderbruggen et al. 2020; Tholen et al. 2022; Armour et al. 2022; Case et al. 2022; Otiashvili et al. 2022). Therefore, in the present study, the insignificant changes in cannabis use may be related to a combination of ongoing health, access, and social gathering concerns across the study period. By extension, it has been reported that many individuals did manage the psycho-social stress of the pandemic with other non-cannabinoid-based coping mechanisms such as increased tobacco, alcohol, and benzodiazepine use (Mezaache et al. 2022; Boehnke et al. 2021; Cousijn et al. 2021).

In addition to the positive association with depression in our LCS model, CUDIT-R scores were negatively associated with the consumption of fruits/vegetables. This is in line with prior research showing that decreased intake of healthy foods is linked with poorer mental health (Esmaillzadeh et al. 2018).

This study has a number of strengths including the use of a prospective cohort to longitudinally assess cannabis use, mental health, and sleep not only before and during stay-at-home measures, but also one-year into the pandemic. Most of the literature on cannabis-use trends during the pandemic have relied on cross-sectional surveys involving retrospective questionnaires to assess use during pre-pandemic/lockdown periods, thus introducing recall and recency bias, whereas surveys in this study did not require such retrospective assessments of health behaviors. Additionally, we measured a number of possible confounds including the use of other substances, as well as diet.

Study limitations include the use of a convenience sample with no known chronic diseases, who initially engaged with the study with an interest in participating in a multiday in-laboratory sleep study, which may reduce overall generalizability. Additionally, all measures are based on self-report which can be subject to bias. This includes our measure for TTB. We are unable to examine total sleep time given the large prevalence of trouble sleeping in our population which likely equates to larger delays to sleep onset and/or an increase of wake after sleep onset. This may have been offset by the use of validated sleep surveys including the PSQI, however, as the PSQI survey was not used for the initial screening prior to COVID-19 it was not included in the follow up surveys.

## Conclusion

The study findings highlight complex relationships between cannabis use, sleep, and mental health during and after COVID-19 stay-at-home orders. While cannabis use frequency declined over time, symptoms of hazardous or disordered cannabis use increased. Sleep trouble was highest during the stay-at-home period and was linked to later cannabis use disorder symptoms, but changes in cannabis use disorder symptoms did not predict changes in sleep trouble. Additionally, worsening sleep trouble was associated with increasing depression and anxiety, emphasizing the interconnected nature of sleep and mental health. These results underscore the need for continued research on the long-term effects of cannabis use and mental health during periods of heightened stress and uncertainty as with COVID-19 stay-at-home orders.

## Supplementary Information

Supplementary Material 1: Fig. 1. Cross-Lagged Panel Model comparing Total Time in Bed on Weekdays with CUDIT-R scores for the three time periods. TTB Weekday = total time in bed weekday as assessed from self-reported in-bed and out-bed times. “CUDIT-R” = Cannabis Use Disorders Identification Test Revised (CUDIT-R) determined at each time point (T0, T1, and T2). Significance determined as a *p* < 0.05 and labeled in the figure with the “*” symbol. Fig. 2. Cross-Lagged Panel Model comparing Total Time in Bed on Weekends with CUDIT-R scores for the three time periods. TTB Weekend = total time in bed weekend as assessed from self-reported in-bed and out-bed times. “CUDIT-R” = Cannabis Use Disorders Identification Test Revised (CUDIT-R) determined at each time point (T0, T1, and T2). Significance determined as a *p* < 0.05 and labeled in the figure with the “*” symbol. Fig. 3. Latent Change Score Model with total time in bed on weekdays assessing health-behavior metric associations between T1 (stay-at-home measures) and T2 (1 yr after stay-at-home measures). “TTB WD” = Total Time in Bed on Weekdays, “Can” = CUDIT-R scores, “Stress” = stress score from perceived stress scale (PSS), “Dep” = depression score from PROMIS questionnaire, "Anx” = anxiety score from PROMIS questionnaire, “Alc” = alcohol use as latent factor score, “FV” = consumption of fruits/vegetables as continuous numeric value. Directional arrows and associated path coefficients are shown for only those comparisons that resulted in significance ( *p* < 0.05 indicated by “*”). Fig. 4. Latent Change Score Model with total time in bed on weekends assessing health-behavior metric associations between T1 (stay-at-home measures) and T2 (1 yr after stay-at-home measures). “Sleep WE” = total time in bed on weekends, “Can” = CUDIT-R scores, “Stress” = stress score from perceived stress scale (PSS), “Dep” = depression score from PROMIS questionnaire, "Anx” = anxiety score from PROMIS questionnaire, “Alc” = alcohol use as latent factor score, “FV” = consumption of fruits/vegetables as continuous numeric value. Directional arrows and associated path coefficients are shown for only those comparisons that resulted in significance ( *p* < 0.05 indicated by “*”).

## Data Availability

The data that support the findings of this study are available from the corresponding author, N.P.B. upon reasonable request.
