# Supplementary figures and images for: Sleep, psychological symptoms, and cannabis use before, during, and after COVID-19 “stay-at-home” orders: a structural equation modeling approach
Source: J Cannabis Res. 2025 Mar 24;7:18. doi: 10.1186/s42238-025-00269-9 (PMC11931863; doi:10.1186/s42238-025-00269-9)

Supplemental Figure 1.

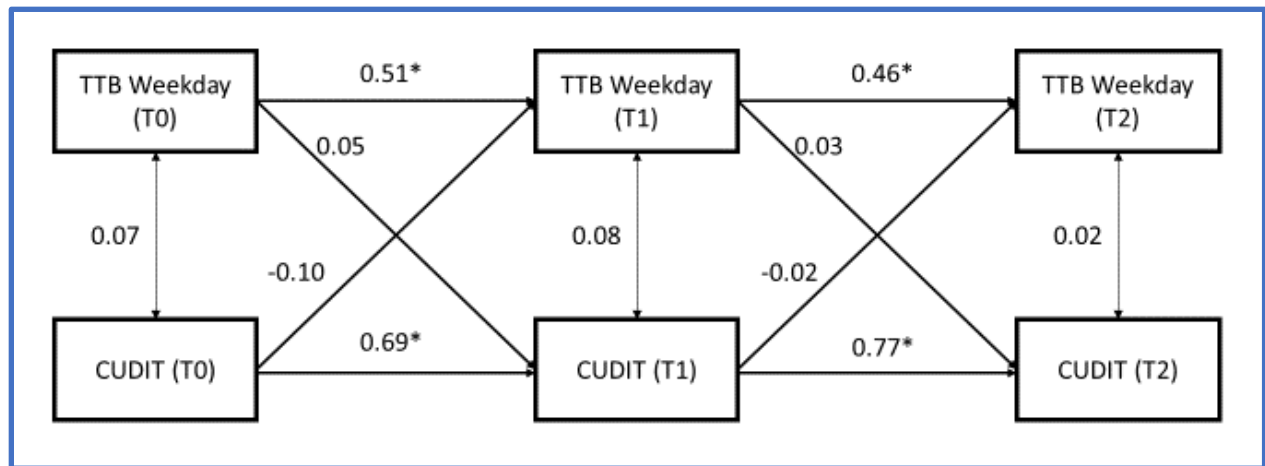

Supplemental Figure 2.

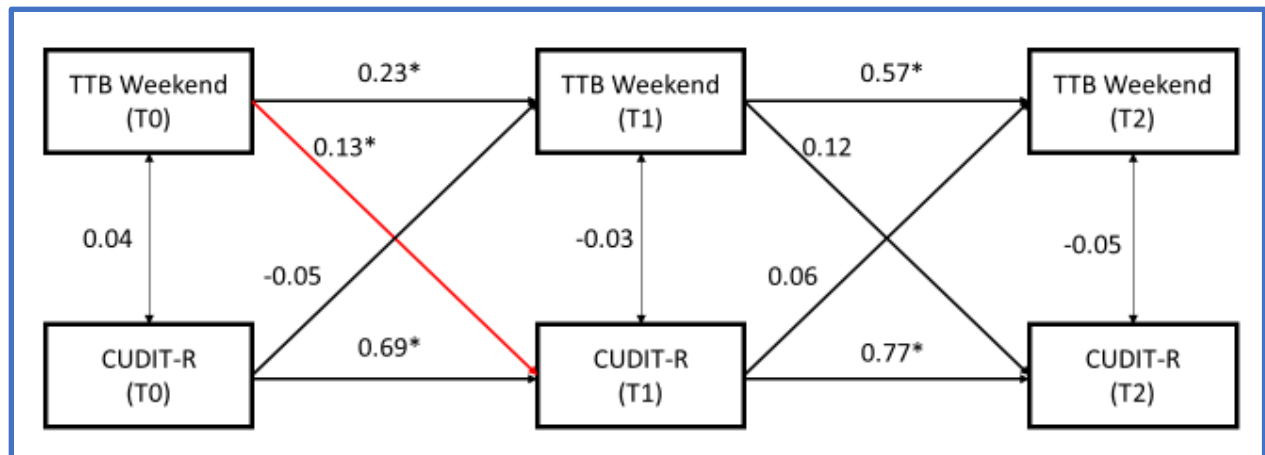

Supplemental Figure 3.

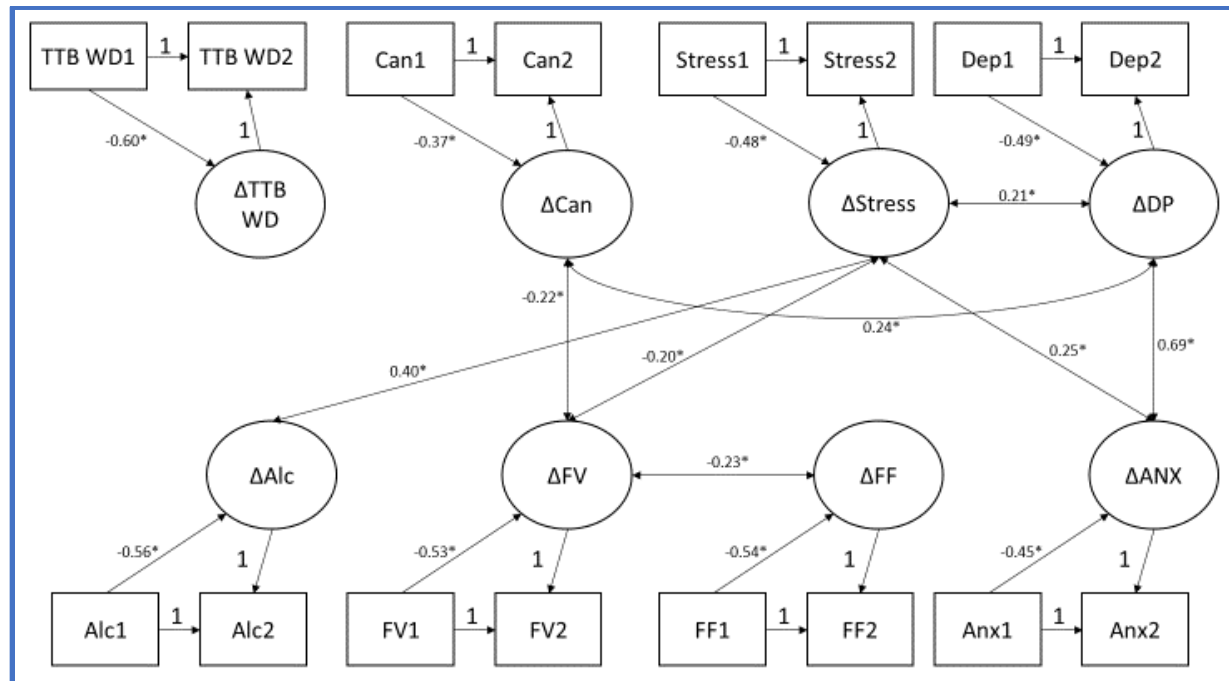

Supplemental Figure 4.

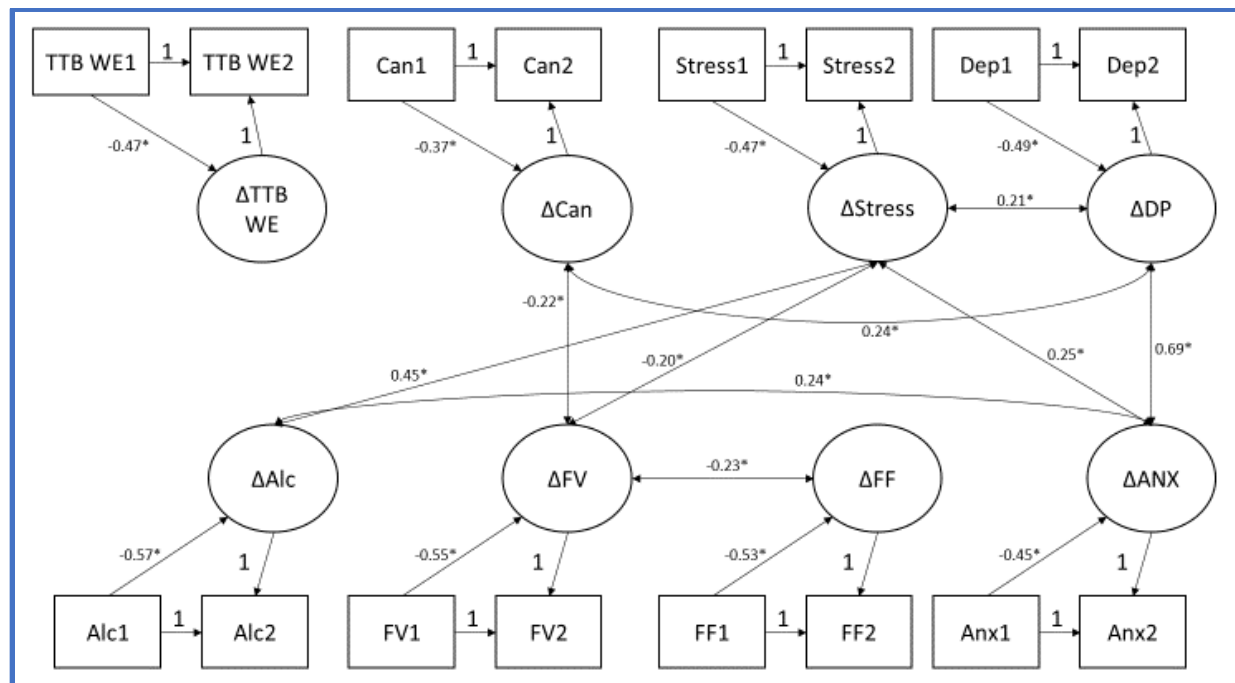

Supplement: Supplementary file 1 — Supplementary Material 1: Fig. 1. Cross-Lagged Panel Model comparing Total Time in Bed on Weekdays with CUDIT-R scores for the three time periods. TTB Weekday = total time in bed weekday as assessed from self-reported in-bed and out-bed times. “CUDIT-R” = Cannabis Use Disorders Identification Test Revised (CUDIT-R) determined at each time point (T0, T1, and T2). Significance determined as a p < 0.05 and labeled in the figure with the “*” symbol. Fig. 2. Cross-Lagged Panel Model comparing Total Time in Bed on Weekends with CUDIT-R scores for the three time periods. TTB Weekend = total time in bed weekend as assessed from self-reported in-bed and out-bed times. “CUDIT-R” = Cannabis Use Disorders Identification Test Revised (CUDIT-R) determined at each time point (T0, T1, and T2). Significance determined as a p < 0.05 and labeled in the figure with the “*” symbol. Fig. 3. Latent Change Score Model with total time in bed on weekdays assessing health-behavior metric associations between T1 (stay-at-home measures) and T2 (1 yr after stay-at-home measures). “TTB WD” = Total Time in Bed on Weekdays, “Can” = CUDIT-R scores, “Stress” = stress score from perceived stress scale (PSS), “Dep” = depression score from PROMIS questionnaire, "Anx” = anxiety score from PROMIS questionnaire, “Alc” = alcohol use as latent factor score, “FV” = consumption of fruits/vegetables as continuous numeric value. Directional arrows and associated path coefficients are shown for only those comparisons that resulted in significance ( p < 0.05 indicated by “*”). Fig. 4. Latent Change Score Model with total time in bed on weekends assessing health-behavior metric associations between T1 (stay-at-home measures) and T2 (1 yr after stay-at-home measures). “Sleep WE” = total time in bed on weekends, “Can” = CUDIT-R scores, “Stress” = stress score from perceived stress scale (PSS), “Dep” = depression score from PROMIS questionnaire, "Anx” = anxiety score from PROMIS questionnaire, “Alc” = alcohol use as l [file 42238_2025_269_MOESM1_ESM.pdf]
